# Efficacy and Mechanism of Active Fractions in Fruit of *Amomum villosum* Lour. for Gastric Cancer

**DOI:** 10.7150/jca.61310

**Published:** 2021-08-20

**Authors:** Jianjun Yue, Shulei Zhang, Bo Zheng, Faisal Raza, Zuhan Luo, Xiaohua Li, Yongyu Zhang, Qu Nie, Mingfeng Qiu

**Affiliations:** 1 School of Traditional Dai-Thai Medicine, West Yunnan University of Applied Sciences; Jinghong, Yunnan 666100, China.; 2 School of Pharmacy, Shanghai Jiao Tong University; Shanghai 200240, China.

**Keywords:** *Amomum villosum* Lour., gastric cancer, active fraction, efficacy, mechanism

## Abstract

Amomi Fructus is the dried ripe fruit of *Amomum villosum* Lour. (*A. villosum*). It is a well-known traditional Chinese medicine widely used to treat gastrointestinal diseases, while the efficacy or mechanism of main components in Amomi Fructus on cancer treatment remains unknown. In this study, volatile oil of *A. villosum* (VOAV), total flavonoids of *A. villosum* (FNAV) and the other residue of *A. villosum* (RFAV) were distilled, extracted and separated as different active fractions of *A. villosum*. The cell toxicity test results indicated that VOAV and FNAV can effectively inhibit the cell growth of MFC cells. Flow cytometry test results confirmed that MFC cells were caused apoptosis after being treated with VOAV, FNAV or RFAV. VOAV, FNAV or RFAV induced MFC cells apoptosis through reactive oxygen species (ROS)-mediated mitochondrial pathway, evident by the increase of endogenous ROS and mitochondrial membrane potential collapse. In addition, FNAV exhibited robust inhibitory effects on MFC tumor growth, and could improve the health status of mice compared to that of mice in 5-FU treated group. To sum up, all the above results suggest that FNAV may be a good candidate for the development of new drugs for the treatment of gastric cancer.

## Introduction

Gastric cancer (GC) is the fifth most common cancer and the third leading cause of cancer-related death worldwide 1. The treatment of GC includes various strategies such as surgical resection, chemotherapy, radiotherapy, immunotherapy and traditional medicine, but the prognosis is still poor 2, 3. Mainly, severe adverse effects and dose-limiting toxicities of chemotherapy treatments are common in clinic 4. In addition, more than 50% of resected GC patients experience recurrence, metastases, and unavoidable resistance to chemo and radiation therapy 5. Nowadays, it is a hotspot for tumor treatment to search for safe and effective antitumor drugs from traditional Chinese medicine 6. Ingredients in them have been found to be useful for the treatment of gastric cancer 7, 8. They are safe and have more biocompatibility as compared to synthetic agents 9, 10.

*Amomum villosum* Lour. (*A. villosum*) is a classic traditional Chinese herbal plant in *Chinese Pharmacopeia* 11. Amomi Fructus, the dry and mature fruit of *A. villosum*, is commonly used to treat gastrointestinal diseases 12-14. The main components in Amomi Fructus are essential oils, saponins, flavonoids, and polysaccharides 15. It is reported that volatile oil and flavonoids in Amomi Fructus may contribute to biological functions, such as anti-inflammation, antimicrobial and anti-nociceptive activities 16, 17.

However, although the biological functions of *A. villosum* has been widely studied in the treatment of gastrointestinal diseases, little attention has been paid to the anti-cancer activity of *A. villosum*. Some Chinese prescriptions containing *A. villosum* such as Additive Xiangsha Liujunzi Decoction result in outstanding effects on GC 18-20. Previous studies have shown that ingredients in *Amomum xanthioides* Wall. ex Baker have anti-gastric cancer activity *in vitro* 21. Therefore, in this study, volatile oil of *A. villosum* (VOAV), total flavonoids of *A. villosum* (FNAV) and the other residue of *A. villosum* (RFAV) were distilled, extracted and separated as different active fractions in *A. villosum*. The cytotoxicity of VOAV, FNAV or RFAV against cancer MFC cells was evaluated by CCK-8 methods *in vitro*. Furthermore, the apoptosis rate, ROS level and mitochondrial membrane potential into the MFC cells were also detected to explore their possible mechanisms. At last, the results of the antitumor activity assay *in vivo* further illustrated the inhibitory effect of FNAV on MFC tumor cell growth.

## Materials and Methods

### Material, Reagents and Animals

Cell Counting Kit-8 (CCK-8), mitochondrial membrane potential assay kit with JC-1, Annexin V-FITC Apoptosis Detection Kit and Reactive Oxygen Species (ROS) assay kit were purchased from Beyotime Biotechnology. RPMI1640 medium, fetal bovine serum (FBS), penicillin and streptomycin were purchased from Thermo Fisher Scientific (Waltham, Massachusetts, USA). 5-fluorouracile (5-FU) was purchased from Macklin Biochemical Technology Company (Shanghai, China). The MFC murine gastric cancer cell line was purchased from Cell Bank of Chinese Academy of Sciences (Shanghai, China) and cultured under recommended conditions.

BALB/c mice were purchased from Shanghai Jiesijie Experimental Animal Co., Ltd. All animal experiments were performed according to the Guidelines for Care and Use of Laboratory Animals of Shanghai Jiao Tong University approved by the Animal Ethics Committee (Number: 202004020 Date: 10 April, 2020).

### Preparation of VOAV, FNAV and RFAV in *A. villosum* Fruit

300 g of* A. villosum* fruit was milled to powder and soaked in 3 L of water for 1 h at room temperature, VOAV was collected by steam distillation for 6 h, then a little water in it was removed with anhydrous sodium sulfate. The VOAV was 3.03 % (mL/g) of the crude drug.

After the distillation of VOAV, a 200-mesh press cloth was used to filter, and filtrate liquor (B) was reserved. Then the residue herb was extracted two times, with 2.4 L of 60 % ethanol and 1 h for each time. 60 % ethanol extracts liquor were filtrated using a 200-mesh press cloth. Then, after being filtrated, 60 % ethanol extracts were condensed until without ethanol inside. Subsequently, concentrated ethanol extracts and filtrate liquor (B) were mixed and concentrated as sample C.

The 750 cm^3^ AB-8 macroporous resin was loaded into the chromatographic column by wet pack. Sample C was loaded into the column, and the solution flowing out of the column was collected at a speed of 1 BV/h as solution D. Then, 2 BV pure water was loaded into the column to remove water-soluble impurity. The solution D and the above eluent were mixed and condensed as RFAV. Subsequently, 2 BV 80 % ethanol was loaded into the column to elute flavonoid until the chromogenic reaction of flavonoid of eluents was negative. The eluent was combined and condensed as FNAV. The content of FNAV was 1.61 % (g/g) of the crude drug and the dry extract yield of RFAV was 4.52 % (g/g) of the crude drug.

### *In vitro* Cytotoxicity Assay

*In vitro* cytotoxicity tests of VOAV, FNAV or RFAV were measured by CCK-8, as described in the manufacturer's protocol. MFC cells were cultured in 96-well plates at a density of 1×10^4^ cells/well, allowed to grow to confluence in 5 % CO_2_ incubator at 37 °C for 24 h. Then, VOAV, FNAV or RFAV at different concentrations (0, 5, 10, 25, 50, 100, 150 μg/mL) were added to each well and then the cells were incubated for 24 h. Then, 10 μL CCK-8 solution was added into each well and incubated at 37 °C for 2 h. Absorbance was measured at 450 nm using a Multimode Reader (Varioskan Flash, Thermo Scientific, USA). The cell viability was calculated as follows:

Cell viability (%) = (OD_experiment_-OD_blank_)/(OD_control_-OD_blank_) × 100%

All assays were repeated three times and the half-maximal inhibitory concentration (IC50) was also calculated by Graphpad Prism software.

### Cell Apoptosis Assays

Cancer cell apoptosis was determined with flow cytometry using a FITC-annexin V/propidium iodide (PI) staining kit. Cells were seeded in 6-well plates at 2×10^5^ per well and incubated for 24 h. The cells were then treated with VOAV, FNAV or RFAV at different concentrations (25, 50, 100 μg/mL) and incubated for 24 h at 37 °C. Then, cells were stained according to the manufacturer's protocol. The cells were analyzed by flow cytometer (LSRFortessa, Becton Dickinson, USA), and the data were analyzed by the Flowjo software (Becton Dickinson, USA).

### Mitochondrial Membrane Potential (MMP) Assay

MFC cells at 1 × 10^6^ cells per well were seeded in 6-well plates and incubated for 24 h with VOAV, FNAV, RFAV at 50 μg/mL or carbonyl cyanide 3-chlorophenylhydrazone (CCCP, as the positive control) at 10 μM, and then washed three times with 2 mL of cold PBS per well. JC-1 was used to stain the cells at 37 °C in the dark for 20 min according to manufacturer's protocol. Then the supernatant was removed and washed two times with 2 mL of JC-1 staining buffer. 2 mL RPMI 1640 medium was added to each well and the cells in 6-well plates were imaged under IXplore Systems (Olympus IX73, Japan). Subsequently, cells were harvested, washed and analyzed by flow cytometry for the ratio of red/green fluorescence intensity.

### Reactive Oxygen Species (ROS) Detection

The changes in intracellular ROS levels were observed with the specific fluorescent probe 2,7-dichloro-dihydrofluorescein diacetate (DCFH-DA). MFC cells were placed in a 6-well plate at a density of 1×10^6^ per well. After 24 h, the medium in the wells was replaced with a medium containing the corresponding concentration of VOAV, FNAV, RFAV or Rosup (as the positive control) at 50 μg/mL and incubated for 24 h. Then the cells were washed three times with 2 mL of cold PBS per well. 10 μM DCFH-DA was used to dye the cells at 37 °C for 20 min in the dark according to the manufacturer's protocol. Then the supernatant was removed and washed three times with 2 mL of RPMI 1640 medium. Finally, the cells were suspended in PBS and the DCFH-DA fluorescence intensity was calculated by flow cytometry.

### *In vivo* Antitumor Test

Female BALB/C mice were injected subcutaneously in the right flank with 3×10^6^ MFC gastric cancer cells per mouse. When tumor volumes reached approximately 60 mm^3^, mice were weighed and randomly divided into five groups. A negative control group was given an equal amount of normal saline by intragastric administration, a positive control group was treated with intraperitoneal injection of 5-FU (2.5 mg/kg per day). VOAV, FNAV or RFAV treated group was intragastrically administrated at 20 mg/kg per day. Drugs were administrated daily by gavage for 14 days. Tumors were collected on Day 20 and the antitumor efficacy and systemic toxicity were assessed by measuring the tumor volume and body weight, respectively. Tumor volume was calculated as the following formula: Tumor volume (V) = a × b^2^/2, in which a and b were the longest and shortest diameter of a tumor measured by vernier caliper.

Tumors, heart, lung, liver, spleen and kidney of mice were collected, fixed with 4 % paraformaldehyde for 24 h, dehydrated by ethanol/acetone and embedded in paraffin. Paraffin-embedded organs were cut at 5 μm thickness, and stained with hematoxylin and eosin (H&E). And then the histological variation was observed under microscope.

### Statistical Analysis

All data were expressed as means ± SD. Statistical significance was evaluated by a t-test. *0.01 < p < 0.05, **0.001 < p < 0.01, ***p < 0.001 were considered statistically significant.

## Results

### Preparation of VOAV, FNAV and RFAV in *A. villosum* Fruit

**Figure 1:**
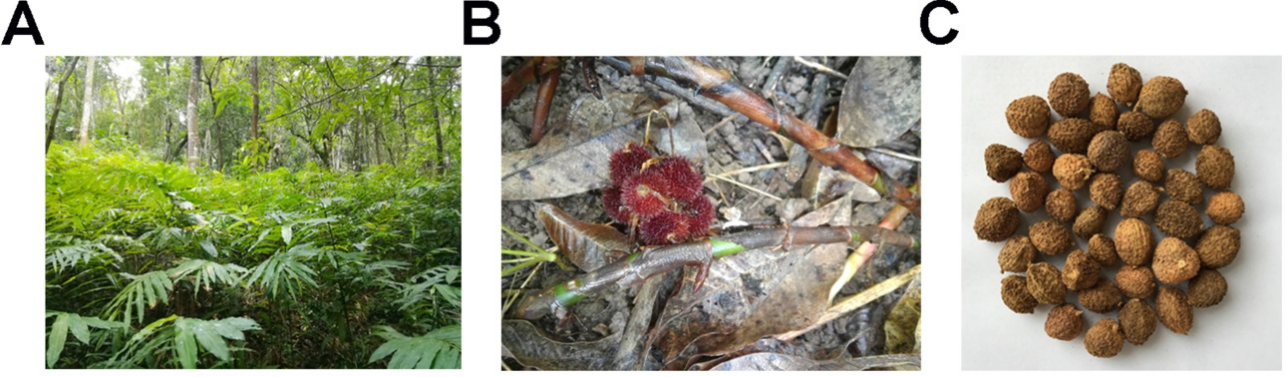
** (A)** Plant of *Amomum villosum* Lour. **(B and C)** Fruit of *Amomum villosum* Lour.

Plants were identified as *A. villosum* by Mengyue Wang, associate professor at Shanghai Jiao Tong University (Fig. 1A). Fruit of *A. villosum* was collected in the Xishuangbanna Dai Autonomous Prefecture of Yunnan Province, China (Fig. 1B and 1C). VOAV, FNAV and RFAV were prepared with the above certain progress. As the result, the yield of VOAV, RFAV and FNAV were 3.03 % (mL/g), 4.52 % (g/g) and 1.61 % (g/g) of the crude drug, respectively.

### *In vitro* Growth Inhibitory Activity

The anticancer activities against MFC cells of VOAV, FNAV or RFAV were evaluated by mouse gastric cancer cell line using CCK-8 methods. MFC cells at 1 × 10^4^ cells/well were seeded in 96-well plates, incubated with VOAV, FNAV or RFAV at various concentrations (0, 5, 10, 25, 50, 100, 150 μg/mL) for 24 h at 37 °C. 5-FU was used as a positive control. The IC_50_ values of VOAV, FNAV or RFAV toward the MFC cell lines are listed in Table 1.

**Figure 2:**
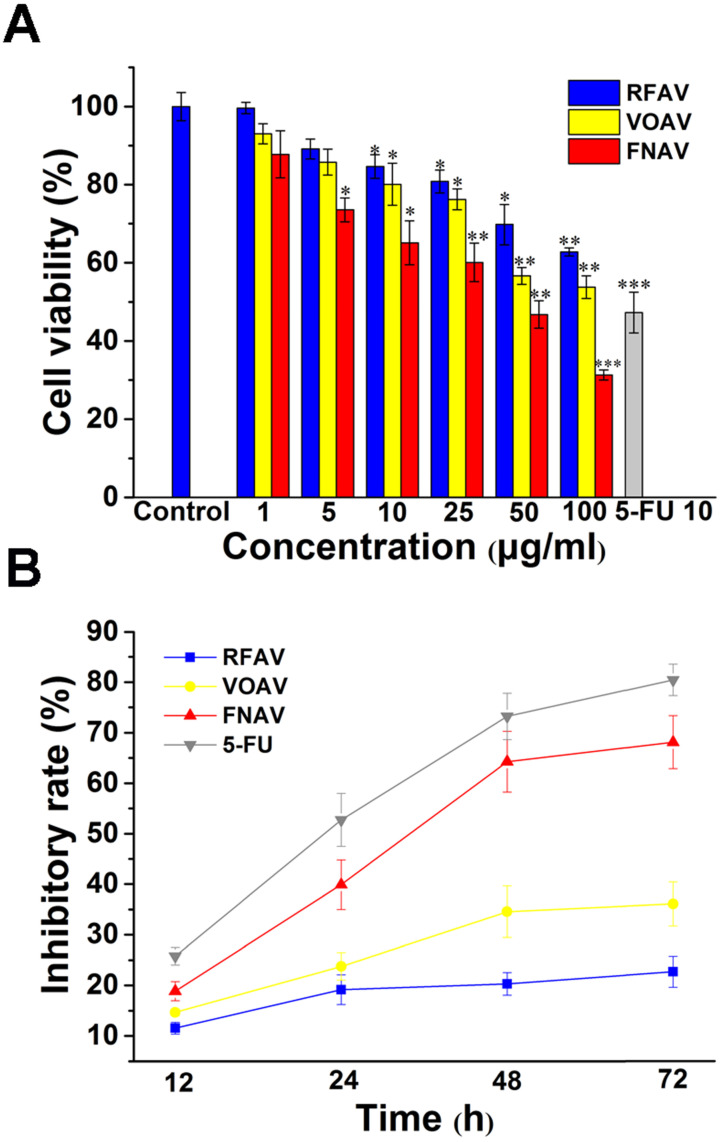
VOAV, FNAV or RFAV inhibited gastric cancer cell proliferation *in vitro*. **(A)** The growth inhibitory effect of VOAV, FNAV or RFAV was measured using the CCK-8 assay. MFC cells were treated with varying concentrations of VOAV, FNAV or RFAV (from 25 to 100 µg/mL for 24 h). **(B)** MFC cells were treated with VOAV, FNAV or RFAV at 25 µg/mL for 96 h. *0.01 < p < 0.05, **0.001 < p < 0.01, ***p <0.001 were considered statistically significant difference from control.

The results showed that treatment with increasing concentrations of VOAV, FNAV or RFAV significantly suppressed the growth of MFC cells in a concentration-dependent manner (Fig. 2A). In Table 1, FNAV showed strong toxicity against MFC cells. 5-FU showed higher cytotoxic activity than VOAV, FNAV or RFAV against MFC cells. The results also suggested that the antitumor activity of the active fractions follows the sequence of FNAV > VOAV > RFAV (Fig. 2B).

### VOAV, FNAV or RFAV Induced Apoptosis of MFC Cells

**Figure 3:**
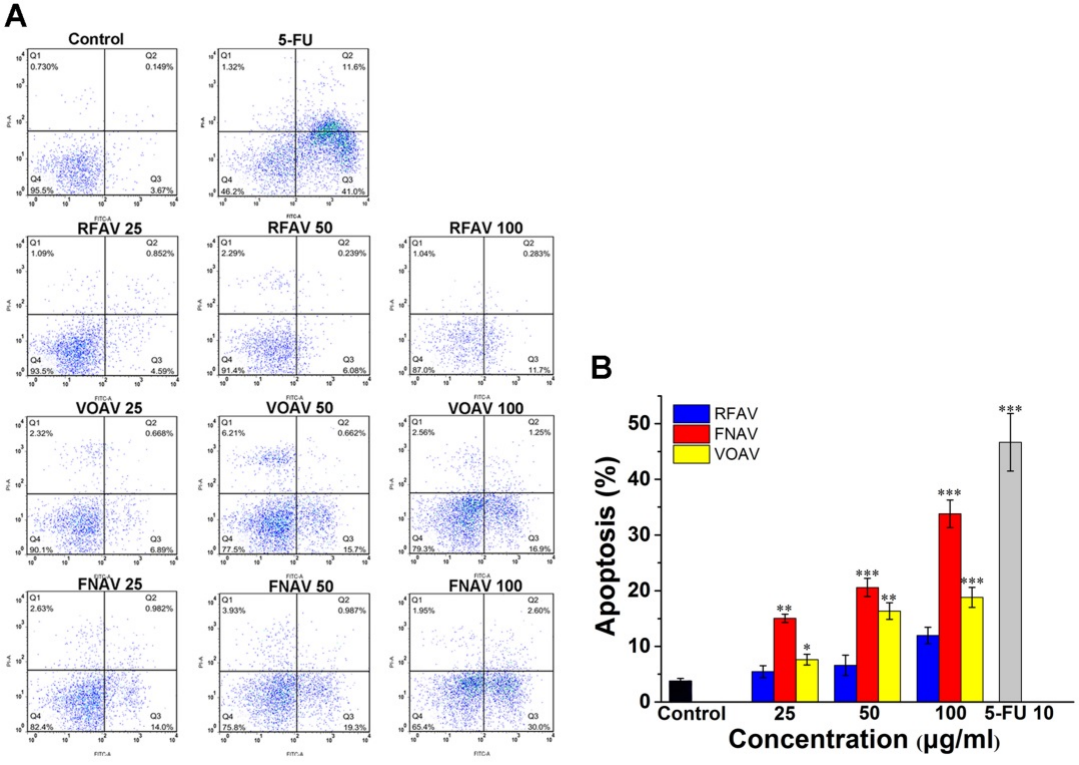
Flow cytometry was used to observe the apoptosis of MFC cells by staining with annexinV-FITC/PI. MFC cells were treated with VOAV, FNAV or RFAV (25, 50, 100 µg/mL) for 24 h. **(A)** Dot plots for group of VOAV, FNAV or RFAV (25, 50, 100 µg/mL). **(B)** Percentage of apoptosis cells, indicated as column histogram. *0.01 < p < 0.05, **0.001 < p < 0.01, ***p <0.001 were considered statistically significant difference from control.

The CCK-8 data indicated that above three active fractions make a momentous difference to reduce the proliferation of MFC cells. To determine the inducing MFC cell growth inhibition features of the three active fractions, flow cytometry analysis was carried out in MFC cells for apoptosis analysis. Annexin V/PI double staining methods were carried out to assess the degree of apoptosis. As shown in Fig. 3A, quantitative results suggested that the early apoptosis rates in MFC cells treated with RFAV, VOAV or FNAV at 25, 50, 100 μg/mL for 24 h were (4.50%, 6.08%, 11.70%), (6.89%, 15.70%, 16.90%), (14.00%, 19.30%, 30.00%) respectively. The results indicated that the active fractions could induce early apoptosis of MFC cells. The results in Fig. 3B exhibited a dose-dependent increase in the degree of apoptosis in annexin V-positive cells, compared to non-treated control group. FNAV induces early apoptosis more strongly than other two fractions.

### Change of Mitochondrial Membrane Potential (MMP)

**Figure 4:**
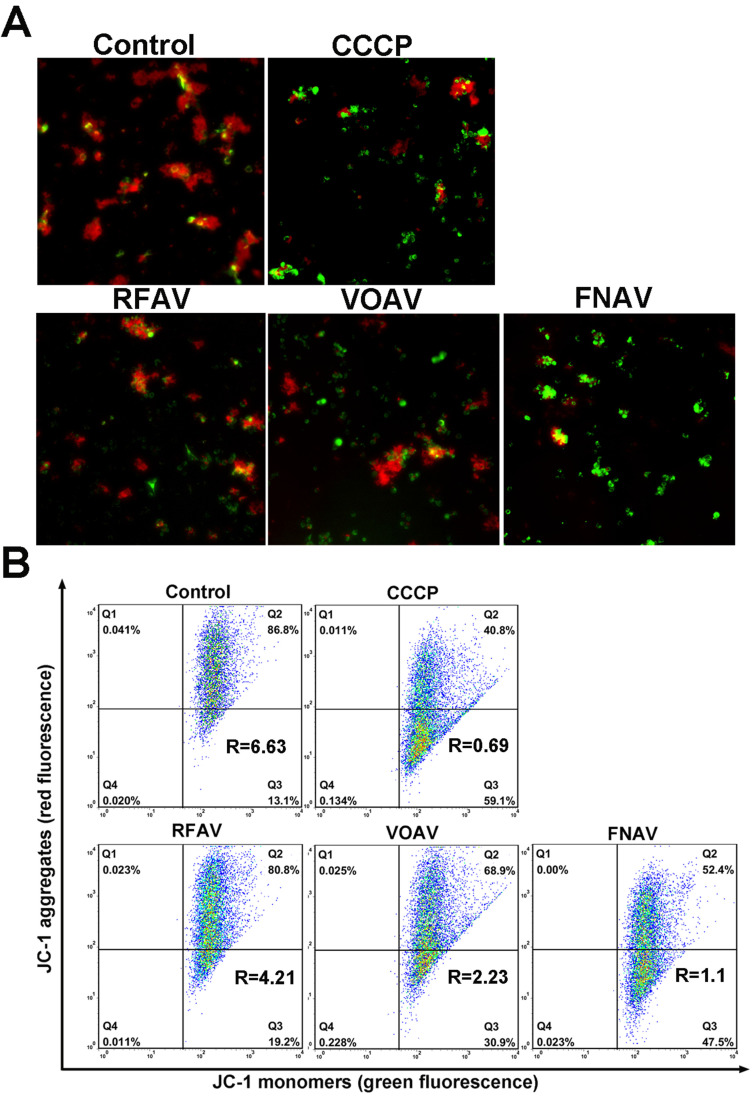
The change in the mitochondrial membrane potential after MFC cells were treated with CCCP (positive control), VOAV, FNAV or RFAV at 50 µg/mL for 24 h. **(A)** MFC cells were detected under a fluorescent microscope. **(B)** The ratio of red/green fluorescence intensity was determined by flow cytometry.

As one of the most important subcellular structures, mitochondria participate in cellular energy generation and apoptotic signaling pathways 22. And the earliest alteration in apoptosis is the reduction of mitochondrial membrane potential. To testify whether VOAV, FNAV or RFAV induce apoptosis in mitochondria- mediated manner, we measured the change of mitochondrial membrane potential by loading MFC cells with fluorescent probe JC-1. As the mitochondrial membrane potential is high, JC-I can accumulate in the mitochondrial matrix and emit red fluorescence. But if it can't accumulate in the mitochondrial matrix, the JC-1 monomer will emit green fluorescence. As depicted in Fig. 4A, the red fluorescence was increasingly superseded by green fluorescence on the addition of VOAV or FNAV into MFC cells clearly. Consequently, the results indicate that VOAV and FNAV can destroy mitochondrial membrane integrity and lower the transmembrane potential in MFC cells.

The relative ratio of red/green fluorescence intensity after the complexes treated MFC cells was determined by flow cytometry, reflecting the mitochondrial depolarization ratio. As shown in Fig. 4B, in the control group and CCCP group (positive control), the red/green rates are 6.63 and 0.69, respectively. After treatment of MFC cells for 24 h, the red/green fluorescence ratios are 4.21 for RFAV, 2.23 for VOAV, 1.10 for FNAV, respectively. According to the above results, we can conclude that FNAV or VOAV exhibited a strong effect to induce mitochondrial membrane potential collapse of MFC cells.

### Intracellular ROS Measurements

**Figure 5:**
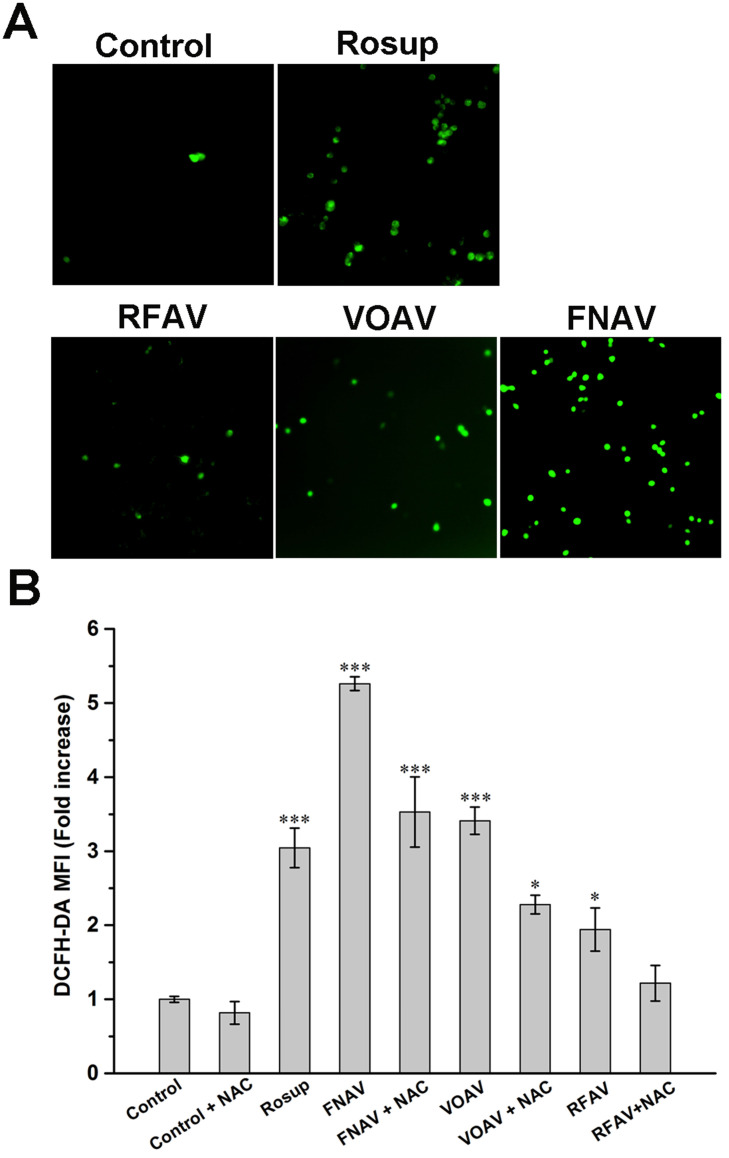
Intracellular ROS was detected in MFC cells. **(A)** MFC cells were detected under a fluorescent microscope. **(B)** The DCF fluorescence intensity was determined by flow cytometry after MFC cells exposed to Rosup and 50 µg/mL of VOAV, FNAV or RFAV in the absence or presence of NAC for 24 h.*0.01 < p < 0.05, **0.001 < p < 0.01, ***p <0.001 were considered statistically significant difference from control.

In order to achieve a deep understanding of the antitumor mechanism of VOAV, FNAV or RFAV, the specific fluorescent probe DCFH-DA was used to detect changes in the reactive oxygen species in MFC cells. As shown in Fig. 5A, a weakly green fluorescence was observed with the control group. MFC cells were incubated with Rosup (positive control), VOAV and FNAV for 24 h, a number of green fluorescence points were found notably. Moreover, flow cytometry was used to further quantified the fluorescence intensity of the treated cells. As shown in Fig. 5B, MFC cells were exposed to VOAV, FNAV or RFAV for 24 h in the absence or presence of NAC, the DCF fluorescent intensity increased 1.94, 3.41 and 5.26 times in comparison with the control group, and the effect of FNAV on the ROS levels was higher than Rosup group. The results suggest that VOAV, FNAV or RFAV can significantly augment the level of cellular reactive oxygen species and FNAV induces the generation of reactive oxygen species more strongly than VOAV and RFAV. The results also demonstrate that NAC inhibits the generation of cellular ROS.

### Inhibition of Tumor Growth by FNAV

**Figure 6:**
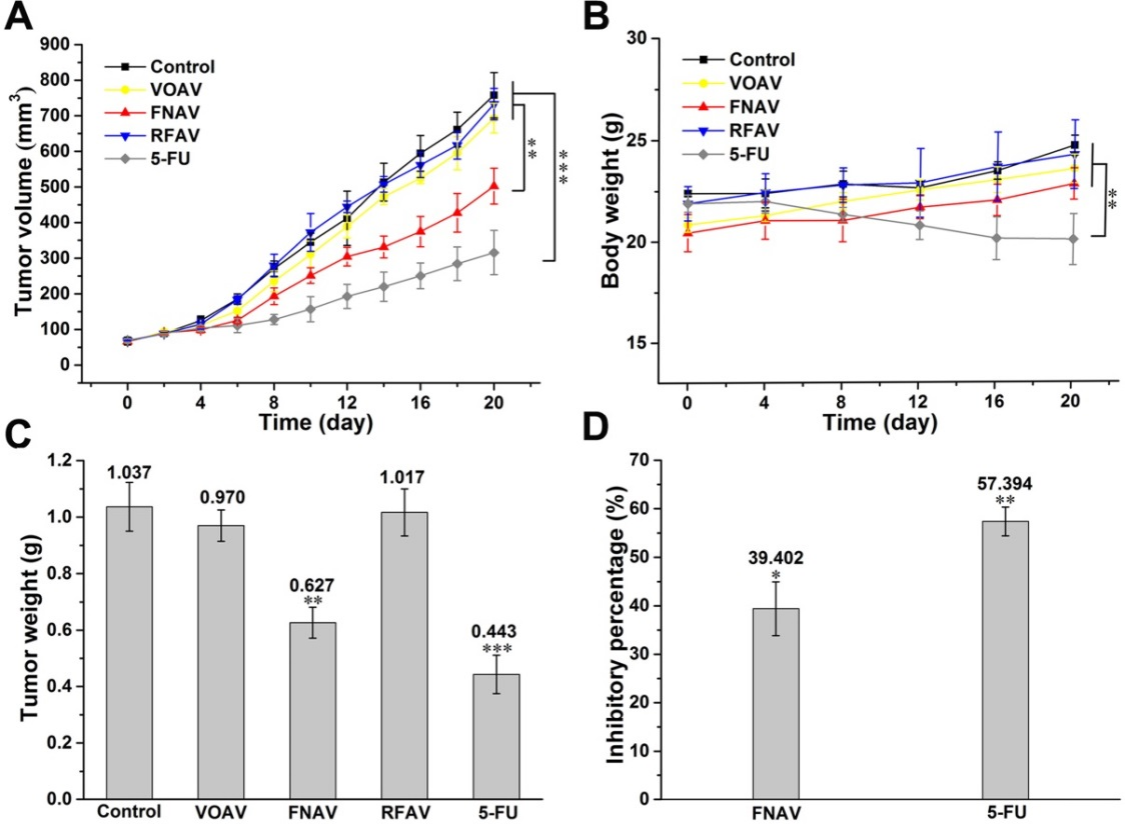
FNAV inhibits gastric tumor growth *in vivo*. **(A)** Tumor volume of mice as a function of time. **(B)** Body weights of the mice over the entire experimental period from the day of injection. **(C)** Tumor weight of each group at the end of the experiment. **(D)** Inhibiting percentage of tumor growth induced by FNAV or 5-FU. *0.01 < p < 0.05, **0.001 < p < 0.01, ***p <0.001 were considered statistically significant difference from control.

The results of a series of experiments *in vitro* confirmed that FNAV showed excellent inhibitory effects on the proliferation against MFC cells. Therefore, a mouse model of gastric cancer was established by the subcutaneous MFC cells inoculation to further evaluate the inhibitory effect of FNAV *in vivo*. Mice were administrated with saline, 5-FU, VOAV, FNAV or RFAV. As shown in Fig. 6A, mice treated with saline exhibited a continued increase in tumor growth up to ~758 mm^3^ at Day 20, while 5-FU treatment significantly decreased tumor volume down to 316 mm^3^. Mice administrated with FNAV showed a significant decrease in tumor volume (502 mm^3^) than mice administrated with VOAV or RFAV (693.5 or 733 mm^3^). Tumors were collected for weight comparison (Fig. 6C).

The body weights of mice were measured to present the health status of mice. 5-FU treatment remarkably induced weight loss, indicating the severe systemic toxicity of 5-FU on mice, which is very common for chemotherapeutic drugs 23. In comparison, FNAV treatment did not induce a decrease of the body weight indicating that FNAV has a better efficacy than 5-FU on improving the health status of mice (Fig. 6B). The inhibiting percentages of tumor growth induced by FNAV or 5-FU are 39.40% and 57.39%, respectively (Fig. 6D). Evidently, FNAV was demonstrated to have better therapeutic effects than VOAV or RFAV on tumor suppression.

**Figure 7:**
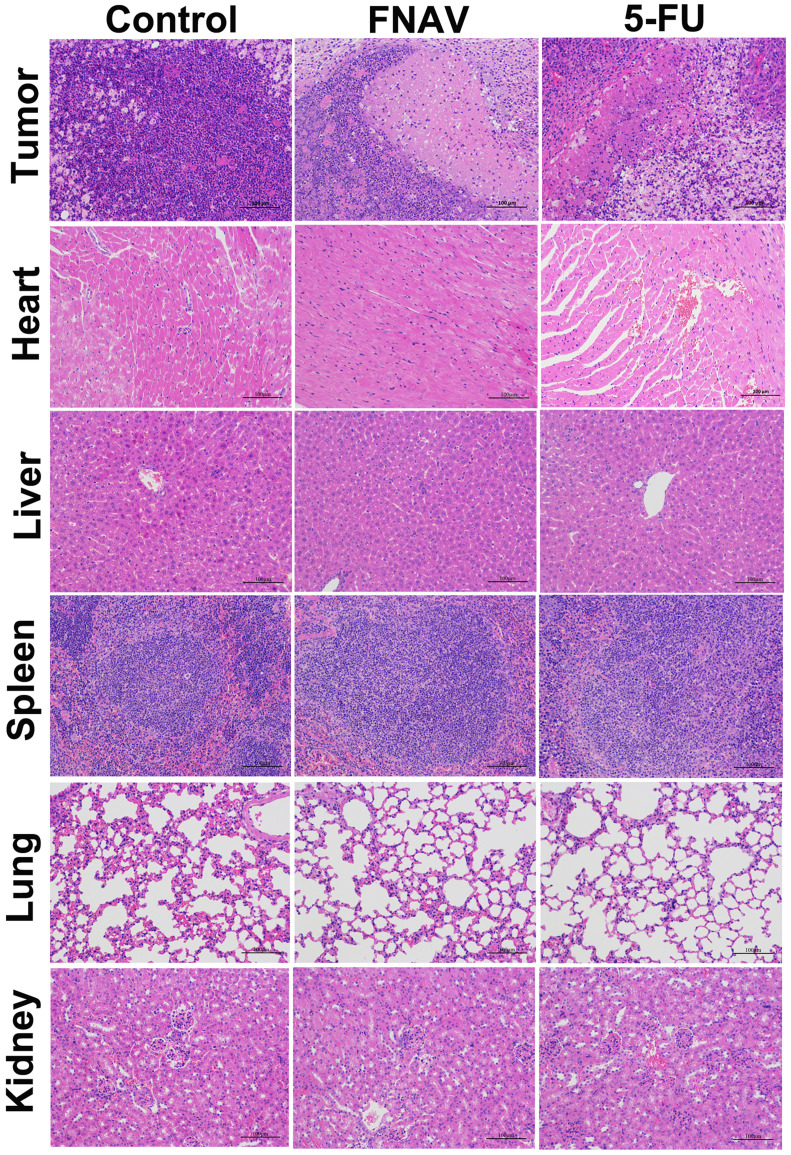
Histological observation of MFC tumors and major organs after the treatment of FNAV or 5-FU. The organs were harvested from MFC tumor-bearing mice at Day 20.

To further evaluate efficacy after treatment with FNAV or 5-FU, the tumors were dissected from mice and sectioned for pathology analysis after 20 days. As shown in Fig. 7, the tightly packed tumor cells interspersed with large amount of stroma were observed in the tumor tissue treated with control group, in which more chromatin and binucleolates were also observed, indicating a rapid tumor growth. The tumor cellularity decreased significantly. And various degrees of tissue necrosis, extensive nuclear shrinkage and fragmentation were observed in the 5-FU treated groups. The necrosis area of tumor of mice in the FNAV treated group was larger than 5-FU treated groups, indicating more efficiency on prevention of cancer cell expansion.

In addition, the representative sections of the main organs including heart, liver, spleen, lung, and kidney were taken at Day 20 from control treated group, FNAV or 5-FU treated group were stained by H&E (Fig. 7). Compared with the control group, histological analysis of the fixed tissues showed that no significant signals could be detected in liver, spleen, lung and kidney derived from animals in the FNAV or 5-FU treated group, such as organ damage, inflammatory response, degeneration, and necrosis. Whereas, heart damage was observed from H&E stained organ slices in the 5-FU treated group due to the observed hyperemia, myocardial fiber breakage and critical pathological changes in cardiac tissue, which was in consistent with the previous report 24, 25. In contrast, the treatment of tumor bearing mice by FNAV did not induce the cardiotoxicity. Therefore, FNAV may become a safer antitumor drug candidate in the future.

## Discussion

In this work, three active fractions in *A. villosum* were prepared and MFC cells were treated with VOAV, FNAV or RFAV at different concentrations of 1-100 μg/mL for 24 h and at 25 μg/mL for 12, 24, 48, 72h, respectively. The results showed that FNAV exhibited considerable anticancer activity with an IC_50_ of 33.21 ± 1.52 μg/mL against the MFC cells. The inhibitory effect on the cells increased along with increase of the drug concentration and prolongation of the action time. The results also suggest that the antitumor activity of the active fractions follows the order of FNAV > VOAV > RFAV on MFC cells *in vitro*.

To further explore the mechanism of the anticancer effects of VOAV, FNAV or RFAV, annexin V-FITC/PI staining was performed, which revealed that VOAV, FNAV or RFAV exerted antitumor effects via induction of apoptosis. Early apoptotic cells, late apoptotic cells, the living cells and necrotic cells can be differentiated by staining with annexinV-FITC/PI, which is a sensitive method to detect early apoptotic cells 10, 26. The apoptotic cells in VOAV group, FNAV group or RFAV group mainly composed of early apoptotic cells indicated that three active fractions induce early apoptosis in MFC cells.

It is well known that the earliest change in apoptosis is the decrease in mitochondrial membrane potential 27. Thus, we tried to figure out if apoptosis induced by three active fractions followed the mitochondrial pathway by estimating the MMP levels and ROS generation. As it turns out, there was an increase in ROS generation and a decline in the MMP levels of MFC cells after treatment with VOAV, FNAV, or RFAV, which ultimately promoted apoptosis.

Previous studies have shown that VOAV prevented the development and progression of intestinal mucositis after chemotherapy, while its inhibiting effect on GC has not been studied 28. Modern pharmacological studies have confirmed that *A. villosum* has the properties of gastrointestinal protection, anti-inflammatory activity, analgesic activity, antidiarrheal activity, antibacterial activity and hypoglycemic activity 29. In comparison, there have been very few studies focusing on antitumor effects of three active fractions in *A. villosum* and related mechanism. It is the first time to demonstrate that active fractions in fruit of *A. villosum* has a better effect on gastric cancer.

In summary, our results showed that FNAV has the most prominent inhibitory effect on MFC cell proliferation. VOAV and FNAV led to a decrease in mitochondrial membrane potential by increasing endogenous ROS production, and then further inducing apoptosis of MFC cells. Finally, the *in vivo* activity evaluation results showed that FNAV has a robust inhibitory effect on MFC tumor growth. All the above results suggested that FNAV might be a promising candidate as an antitumor agent against GC.

## Figures and Tables

**Table 1:** IC_50_ Values of VOAV, FNAV and RFAV against MFC cells

Active Fractions	IC_50_ (μg/mL)
VOAV	98.89 ± 3.37
FNAV	33.21 ± 1.52
RFAV	>100
